# Endochin-Like Quinolones Exhibit Promising Efficacy Against *Neospora Caninum in vitro* and in Experimentally Infected Pregnant Mice

**DOI:** 10.3389/fvets.2018.00285

**Published:** 2018-11-19

**Authors:** Nicoleta Anghel, Vreni Balmer, Joachim Müller, Pablo Winzer, Adriana Aguado-Martinez, Mona Roozbehani, Sovitj Pou, Aaron Nilsen, Michael Riscoe, J. Stone Doggett, Andrew Hemphill

**Affiliations:** ^1^Vetsuisse Faculty, Institute of Parasitology, University of Bern, Bern, Switzerland; ^2^Department of Parasitology, School of Medicine, Iran University of Medical Sciences, Tehran, Iran; ^3^VA Portland Health Care System Research and Development Service, Portland, OR, United States

**Keywords:** *Neospora*, *Toxoplasma*, endochin-like quinolones, mitochondrion, cytochromeb, electron microscopy, vertical transmission, mouse model

## Abstract

We report on the efficacy of selected endochin-like quinolones (ELQs) against *N. caninum* tachyzoites grown in human foreskin fibroblasts (HFF), and in a pregnant BALB/c mouse model. Fourteen ELQs were screened against transgenic *N. caninum* tachyzoites expressing β-galactosidase (Nc-βgal). Drugs were added concomitantly to infection and the values for 50% proliferation inhibition (IC_50_) were determined after 3 days. Three compounds exhibited IC_50_ values below 0.1 nM, 3 ELQs had IC_50_s between 0.1 and 1 nM, for 7 compounds values between 1 and 10 nM were noted, and one compound had an IC_50_ of 22.4 nM. Two compounds, namely ELQ-316 and its prodrug ELQ-334 with IC_50_s of 0.66 and 3.33 nM, respectively, were previously shown to display promising activities against experimental toxoplasmosis and babesiosis caused by *Babesia microti* in mice, and were thus further studied. They were assessed in long-term treatment assays by exposure of infected HFF to ELQs at 0.5 μM concentration, starting 3 h after infection and lasting for up to 17 days followed by release of drug pressure. Results showed that the compounds substantially delayed parasite proliferation, but did not exert parasiticidal activities. TEM of drug treated parasites detected distinct alterations within the parasite mitochondria, but not in other parasite organelles. Assessment of safety of ELQ-334 in the pregnant mouse model showed that the compound did not interfere in fertility or pregnancy outcome. In *N. caninum* infected pregnant mice treated with ELQ-334 at 10 mg/kg/day for 5 days, neonatal mortality (within 2 days *post partum*) was found in 7 of 44 pups (15.9%), but no postnatal mortality was noted, and vertical transmission was reduced by 49% compared to the placebo group, which exhibited 100% vertical transmission, neonatal mortality in 15 of 34 pups (44%), and postnatal mortality for 18 of the residual 19 pups during the 4 weeks follow-up. These findings encourage more research on the use of ELQs for therapeutic options against *N. caninum* infection.

## Introduction

*Neospora caninum* is an obligate intracellular coccidian parasite, which is closely related to *Toxoplasma gondii*, and which until 1988 had been misdiagnosed as such (1). While the overall shape, morphology and the fact that a large panel of animals can be infected with *N. caninum* are features that it shares with *Toxoplasma*, there are also some clearly distinct biological characteristics that distinguish the two species. For instance, canids such as dogs, dingoes, gray wolves, and coyotes are known to act as definitive hosts for *N. caninum* (2–5), while for *Toxoplasma* this role is taken over exclusively by felids. *N. caninum* can infect a variety of intermediate hosts, including economically important farm and food animals (6), but there is no evidence that neosporosis could be zoonotic (1). This is in stark contrast to *Toxoplasma*, which besides being an economically important pathogen, also infects up to one third of the human population, and can cause serious disease in immunodeficient persons and/or upon primary infection during pregnancy with serious consequences for the fetus (7).

Economically, *N. caninum* is most relevant in cattle, in that it can cause repeated abortions, stillbirths and birth of weak calves. This happens either upon primary infection during pregnancy (exogenous transplacental transmission), or through recrudescence in chronically infected dams caused by immunomodulation during pregnancy (endogenous transplacental transmission) (8, 9). In spite of considerable investments, there is currently no commercially available vaccine on the market (10). In the past, drug treatment has not been envisaged as an interesting approach to combat neosporosis, and due to the need of repeated treatments and the potential residues that could remain in meat and milk products from animals destined for consumption, drugs were regarded as economically non-viable. However, the inherent difficulties in generating an efficient vaccine for limiting the effects of neosporosis, most notably in cattle, has sparked the interest for potential treatment options.

An increasing number of compounds, repurposed from older drugs, have been shown to exhibit a potential for drug treatment of animals infected with *N. caninum* [reviewed in Hemphill et al. (11)]. Many of these repurposed drugs and their more recently synthesized analogs were developed for the treatment of malaria, caused by the apicomplexan parasites *Plasmodium falciparum*. These compounds have been greatly improved in terms of high efficacy and specificity, low cytotoxicity, and favorable pharmacokinetic properties, and drug repurposing has demonstrated interesting activities not only against *Plasmodium*, but also other protozoan parasites and also helminths (12, 13).

One of the most interesting compounds is endochin which was proven to have a potent action against liver and erythrocytic stages of an avian malaria model. Similar to other quinolones such as atovaquone and buparvaquone, endochin inhibits the cytochrome *bc*_1_ complex of the mitochondrial electron transport chain of *Plasmodium* (14). Cytochrome *bc*_1_ facilitates the transfer of electrons from ubiquinol to cytochrome *c* and contains both oxidative (Q_o_) and reductive (Q_i_) catalytic sites. The anti-malarial drug atovaquone is a classical Q_o_ site inhibitor (15). More recently, novel endochin analogs with a diphenylether side chain at the third position, and various substitutions at position 5, 6, or 7 of the quinolone ring were synthesized. These include ELQ-300 and ELQ-316, which confer Q*i* site inhibition (16, 17) and ELQ-400 (18), which is an unusual inhibitor as it targets both Q_o_ and Q_i_ sites of cytochrome *bc*_1_ of *S. cerevisiae* and potentially other organisms (19). Structure-activity relationship studies performed by Stickles et al. (18) and McConnell et al. (20) showed that differential binding to either the Q_o_ or Q_i_ site in *P. falciparum* and *T. gondii* cytochrome *bc*_1_ is dependent on subtle changes in the structure of ELQs mediated by various substitutions at different positions of the quinolone ring.

Due to poor aqueous solubility and high crystallinity, ELQ analogs exhibit only limited oral absorption (21). To solve this problem, prodrugs for the compounds with good efficacy *in vitro* were created, which contain a carbonate ester pro-moiety that disrupts crystallinity and increases oral bioavailability. The prodrug is metabolized after administration and releases the active compound, thus leading to increased exposure and therefore improved efficacy (21, 22). One of the ELQs with excellent efficacy against *T. gondii* is ELQ-316, a Q_i_ site inhibitor (23), exhibiting *in vitro* IC_50_ values below 1 nM, and *in vivo* affecting not only tachyzoites, but also tissue cysts (16). ELQ-316 and its prodrug ELQ-334 also exhibited excellent activities in treating *Babesia microti* infections in immunodeficient mice (24).

In this study, we assessed the *in vitro* activities of 14 distinct ELQ analogs against a transgenic *N. caninum* strain expressing beta-galactosidase. We then focused on ELQ-316 and its prodrug ELQ-334 and studied the effects of these compounds in terms of parasiticidal vs. parasitostatic activity, ultrastructural alterations, and assessed the safety and *in vivo* efficacy of ELQ-334 in the pregnant neosporosis mouse model.

## Materials and methods

### Tissue culture media, biochemicals, and compounds

If not stated otherwise, cell culture media were purchased from Gibco-BRL (Zurich, Switzerland) and biochemical reagents were obtained from Sigma (St. Louis, MO, USA). Kits for molecular biology were purchased from Qiagen (Hilden, Germany). The endochin-like quinolones (ELQs) were synthesized as previously described (20), identified by proton nuclear magnetic resonance (^1^H NMR), and determined to be >95% pure by reversed-phase high-performance liquid chromatography (HPLC). For *in vitro* studies, the compounds were stored as 20, 10 mM, or 100 μM stock solution in dimethyl sulfoxide (DMSO) at −20°C. For *in vivo* experiments, ELQ-334 was suspended in corn oil and administered to the mice by oral gavage.

### Cell culture and parasites

Human foreskin fibroblasts (HFF), Vero cells and Marc cells were cultured and maintained as described earlier (25, 26). Tachyzoites of the *N. caninum* isolate Nc-Spain7, and transgenic *N. caninum* strain containing a beta-galactosidase reporter strain (Nc-βgal, with Nc-1 background) were cultured either in Vero cells, Marc cells or HFF, and were maintained and prepared for infections as described before (25–27).

### Cytotoxicity assessments

The toxicity of ELQs against host cells was assessed as previously described (28, 29). Briefly, HFF were grown to confluence in 96 well-plates. After removal of the medium by aspiration, cells received 200 μl fresh medium containing either 2, 1, 0.5, 0.25, 0.125, 0.062, and 0.031 μM of each ELQ, respectively. Non-treated HFF and DMSO controls were included. Plates were kept in an incubator at 37°C and 5% CO_2_ for 3 days. Subsequently, medium was discarded from the well-plates by aspiration, and cells were washed once with PBS. The resazurin stock solution was diluted 1:200 in PBS, and 200 μl were added into each well. Plates were read at λ excitation 530 nm and λ emission 590 nM at the EnSpire® multimode plate reader. Fluorescence was measured different timepoints. Relative fluorescence units were calculated from timepoints with linear increase.

### *In vitro* proliferation measurements employing nc-βgal tachyzoites

Primary screening of the 14 ELQs assessed in this study (see Table 1) was performed using the transgenic Nc-βgal strain cultured in HFF as described earlier (28, 29). HFF monolayers were cultured in 96-well plates, and infected with 10^3^ Nc-βgal tachyzoites per well in the presence of ELQs (stock solutions of 100 μM in DMSO), prepared in serial dilutions in the following concentrations: 200, 20, 2, 0.2, 0.02, 0.002, and 0.0002 nM in medium, respectively. The stock solution, as well as, the medium were heated up to 37°C prior to use. After 3 days of culture in the presence of the drugs, wells were washed once with 200 μl PBS, and 90 μl PBS containing 0.05% Triton X-100 solution was added to each well. After addition of 10 μl of 5 mM chlorophenolred-β-D-galactopyranoside dissolved in PBS, the absorption shift was measured at a 570 nM wavelength at various timepoints on a VersaMax multiplate reader (Bucher Biotec, Basel, Switzerland). IC_50_s were calculated after the logit-log transformation of relative growth and subsequent regression analysis by use of the corresponding software tool contained in the Excel software package (Microsoft, Seattle, WA).

**Table 1:** IC_50_ values of ELQ compounds as determined by *N. caninum* and *T. gondii* transgenic strains expressing β-galactosidase.

**Code**	**MW**	**Structure**	**LogP**	***Nc*IC_50_ (nM)**	***Tg*IC_50_ (nM)^a^**
Endochin	287.4	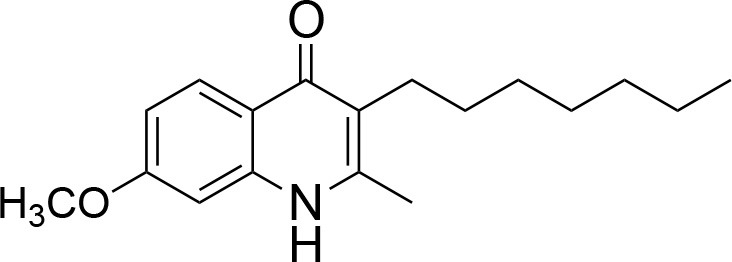	3.35	0.75	0.07
ELQ-432	321.8	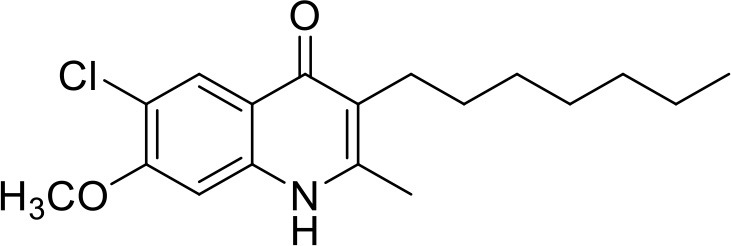	3.91	22.45	43
ELQ-433	305.4	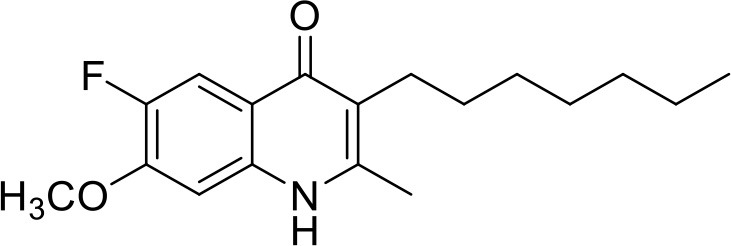	3.51	4.67	3.8
ELQ-127	257	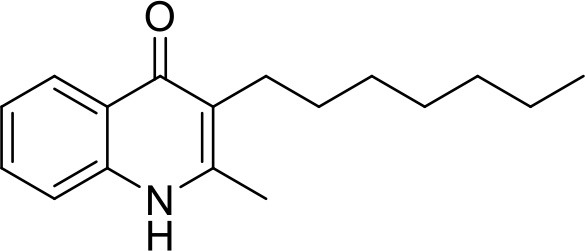	3.5	2.45	2.8
ELQ-136	275.4	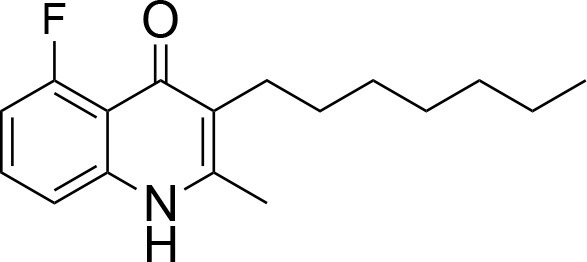	3.63	0.02	0.13
ELQ-121	293.3	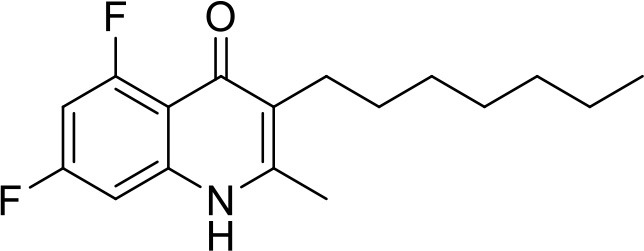	3.79	0.08	0.006
ELQ-436	313.5	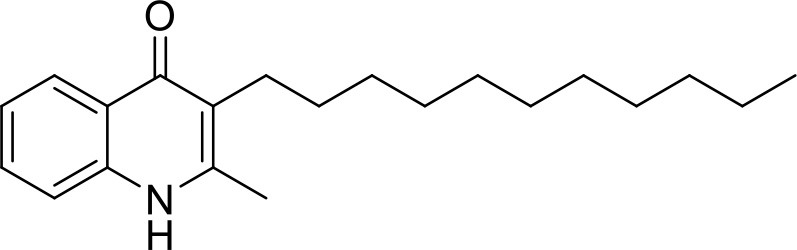	5.15	1.04	0.68
ELQ-434	377.9	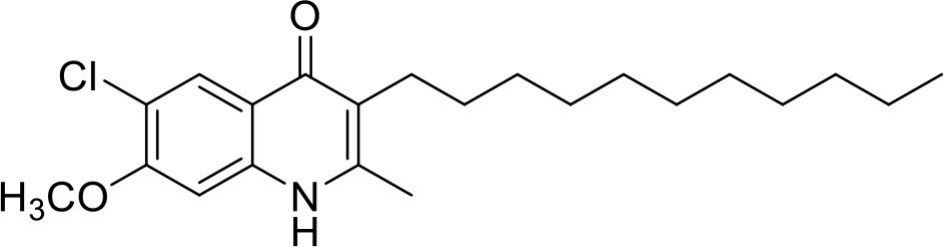	5.58	3.81	11
ELQ-437	349.5	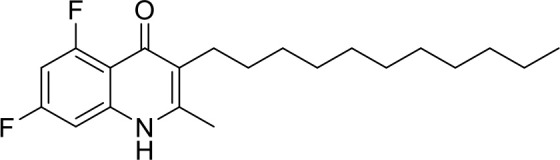	5.46	0.09	0.003
ELQ-435	361.5	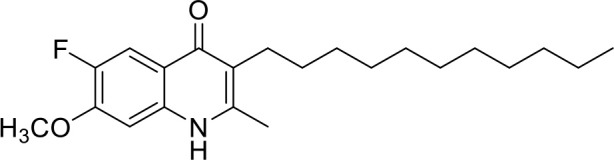	5.18	2.01	0.24
ELQ-300	475.8	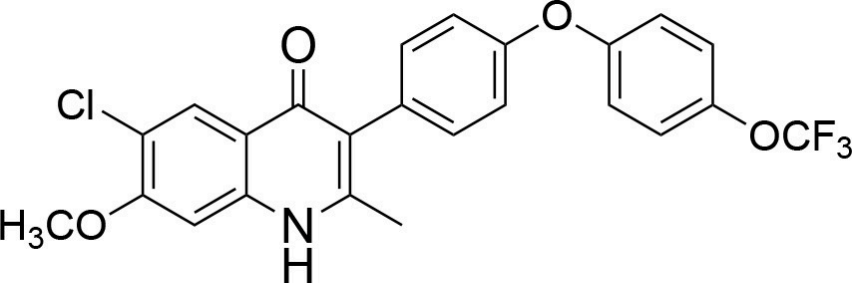	5.66	0.33	25
ELQ-316	459.4	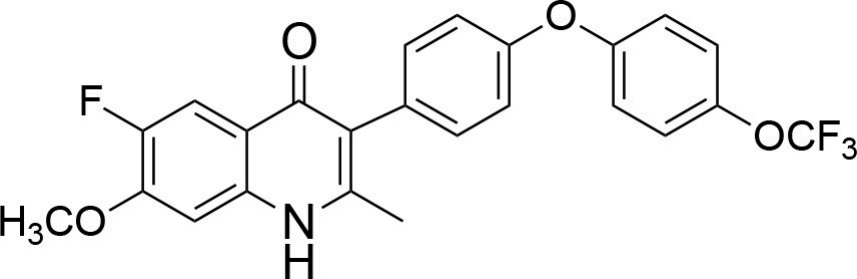	5.26	0.66	0.35
ELQ-271	411	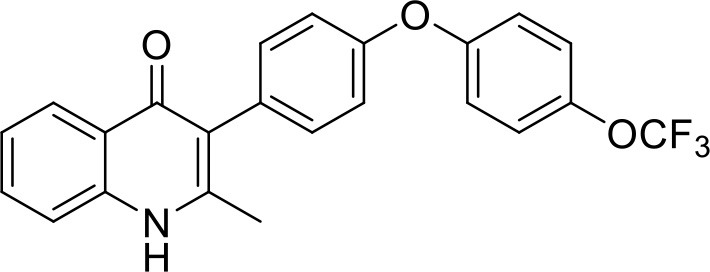	5.22	2.75	5.6
ELQ-334	531.4	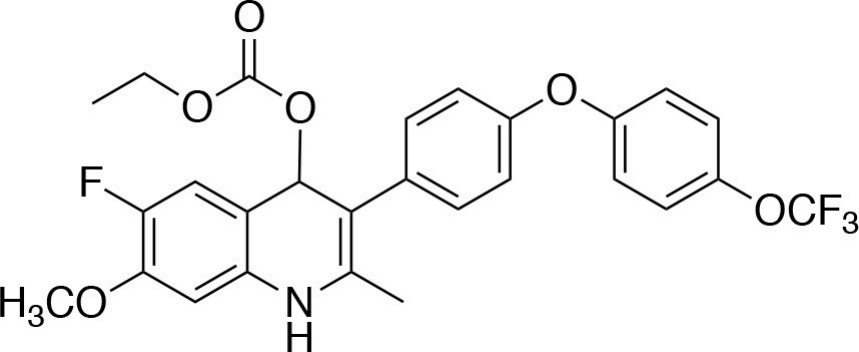	8.1	3.33	ND
ELQ-437	349.5	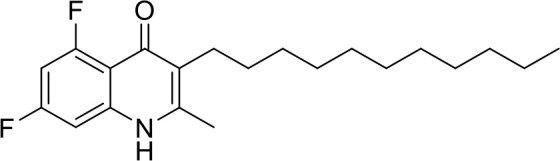	5.46	0.09	0.003

a *Indicates results reported in McConnell et al. (20); ND, not done*.

### *In vitro* proliferation measurements employing nc-spain7 tachyzoites employing quantitative real-time PCR

HFF were grown in 6-well plates until a confluent monolayer was formed. Just prior to infection, ELQ-316 and ELQ-334 were added at concentrations of 200, 20, 2, 0.2, 0.02, 0.002, and 0.0002 nM. Controls received the corresponding amounts of DMSO. HFF were then infected with 3 × 10^5^ freshly purified tachyzoites of *N. caninum* Nc-Spain7. After 4 days, cells were collected with a cell scraper, centrifuged, and washed in PBS. DNA purification was performed employing the DNeasy blood and tissue kit (Qiagen, Basel, Switzerland) according to the standard protocol suitable for animal cells. *N. caninum* parasite load was determined by real-time PCR as previously described (30). IC_50_s were calculated as described above.

### Long-term treatment assays

To assess the effects of extended treatments with ELQs, 5 × 10^5^ HFF were grown to confluence in T25 flasks and were then infected with 1 × 10^6^ Nc-Spain7 tachyzoites for 3 h, washed 2 times with HBSS (Hanks balanced salt solution), and were then exposed to 500 nM ELQ-316 or ELQ-334 for periods of either 3, 6, 9, 12, 14, or 17 days, with medium changes and addition of fresh medium containing the compounds every 3 days. Following treatments, cultures were washed twice with HBSS and were further maintained in medium devoid of drugs. Regrowth of parasites was monitored on a daily basis by light microscopy, until plaques and proliferating parasites were visible, for a maximum period of 3 weeks after drug removal.

### Transmission electron microscopy (TEM)

TEM was carried out essentially as described (25). Briefly, 5 × 10^5^ HFF were grown in T25 flask for 96 h, and were infected with 1 × 10^6^ *N. caninum* Nc-Spain7 tachyzoites. After 4 h, the medium and non-invaded parasites were removed, flasks were washed twice with medium, and infected cultures were maintained at 37°C/5% CO_2_, for 48 h. Subsequently, 1 μM of ELQ-316 and ELQ-334 were added, and after 6, 12, 24, 48, or 72 h, the medium was discarded and the flasks were washed twice with 0.1 M Na-Cacodylate buffer (pH 7.3), and fixed in 2% glutaraldehyde in 0.1 M Na-Cacodylate buffer for 10 min at room temperature. Then, infected monolayers were carefully removed using a cell scraper, transferred into an Eppendorf tube and fixation was continued for 2 h. The specimens were post-fixed in 2% OsO_4_ in cacodylate buffer washed in water, followed by immersion in Uranylless solution (Electron Microscopy Science, Hatfield PA, USA) for 20 min. Following stepwise dehydration in a graded series of ethanol, specimens were embedded in Epon-812 resin, with three changes of epoxy resin every 10–12 h. Following polymerization at 60°C, ultrathin sections were cut using an ultramicrotome (Reichert and Jung, Vienna, Austria), and were placed onto 200 mesh nickel grids (Plano GmbH, Marburg, Germany). Following staining with Uranylless and lead citrate, specimens were viewed on a CM12 transmission electron microscope operating at 80 kV.

### Immunofluorescence labeling of ELQ treated tachyzoites

HFF monolayers were grown to 50% confluency on glass coverslips in 24-well plates and infected with 3 × 10^4^ Nc-Spain7 tachyzoites. After 3 h, specimens were exposed to 1 μM ELQ-316 and ELQ-334 and the culture was continued for a maximal duration of 14 days. At different timepoints, the coverslips were washed once in PBS, followed by fixation and preparation for immunofluorescence as previously described (25, 31, 32). The following primary antibodies were used: (i) anti-IMC1, a rabbit polyclonal antibody directed against the inner membrane complex (IMC1), kindly provided by Prof. Dominique Soldati-Favre, University of Geneva, diluted 1:500; (ii) anti-SAG1, a mouse monoclonal antibody directed against the major immunodominant tachyzoite surface antigen1 (33) diluted 1:1000. Primary antibodies were applied for 20 min at room temperature in PBS with 0.3% bovine serum albumin (BSA). After four washes in PBS, the corresponding secondary antibody conjugates (anti-mouse fluorescein isothiocyanate [FITC] or anti-rabbit Texas Red [Sigma-Aldrich]) were applied at a dilution of 1:300 in PBS with 0.3% BSA. After four washes in PBS, specimens were incubated for 3 min in 4′,6-diamidino-2-phenylindole (DAPI) diluted 1:300 in PBS with 0.3% BSA, washed again for 4 times in PBS, and mounted in Vectashield mounting medium (Vector Laboratories, Burlingame, CA, USA). All specimens were viewed on a Nikon Eclipse E800 digital confocal fluorescence microscope. Processing of images was performed using the OpenLab 5.5.2 software (Improvision, PerkinElmer, Waltham, MA, USA).

## Ethics statement

All protocols involving animals were approved by the Animal Welfare Committee of the Canton of Bern under the license BE115/14. Animals were handled in strict accordance with practices made to minimize suffering. Female and male BALB/c mice, 8 weeks of age were purchased from a commercial breeder (Charles River, Sulzberg, Germany), and were maintained in a common room under controlled temperature and a 14 h/10 h light cycle, with food accessible *ad libitum*. All procedures were carried according to the guidelines set up by the animal welfare legislation of the Swiss Veterinary Office.

### Assessment of potential effects of ELQ-334 on pregnancy outcome in non-infected BalB/c mice

Twelve BALB/c female and 6 male mice, 8 weeks of age (purchased from Charles River, Germany) were used. two groups of six mice were prepared (2 females per cage, 3 cages per group), one ELQ-334 treatment group, and one placebo-treatment group. Females were oestrus-synchronized by the Whitten effect and were mated for three nights by housing 1 male with 2 females (27, 34, 35). After 3 days, males were removed from the cages. Treatments with ELQ-334 were performed from day 9–13 post-mating, calculated from the first day of mating. Prior to the treatment, ELQ-334 was suspended in corn oil, at 10 mg/kg body weight in 100 μl inoculation volume administered by gavage. The placebo group received the equal volume of corn oil only. The weight of each mouse was measured several times during the pregnancy period to determine the number of pregnant animals and to detect possible abortions (which would cause rapid weight loss). On day 18 post-mating, pregnant females were separated into individual cages, where they gave birth on days 20–22 post-mating. Live and stillborn pups were counted, and dead pups were removed. Data on the number of female mice that became pregnant, litter size (number of delivered pups per dam), stillborn mice (number of full-term dead pups from birth until day 2 p.p.); post-natal mortality (number of dead pups from day 3 p.p to the end of the experiment) and clinical signs of treated mice were recorded. Dams and live pups were monitored at least until 2 weeks after birth to rule out possible adverse effects due to treatments. At the end of the experiment, pups were euthanized by decapitation. Adult mice were euthanized in a euthanasia chamber using isoflurane and CO_2_.

### Assessment of ELQ-334 treatment efficacy in non-pregnant and pregnant BALB/c mice experimentally infected with Nc-Spain7

Twenty-eight BALB/c female and 14 male mice, 8 weeks of age and provided by Charles River, were maintained as described above. Two groups of 14 mice were prepared (2 females per cage, 7 cages per group). After mating (see above), all mice were infected with 10^5^ Nc-Spain7 tachyzoites suspended in 100 μl medium by subcutaneous neck injection. Following infection and for the duration of the entire experiment, mice were inspected daily for clinical signs (ruffled coat, apathy, hind limb paralysis) and categorized according to a score sheet approved by the local authorities.

The ELQ-334 treatment group received ELQ-334 formulated in corn oil at 10 mg/kg body weight/day for 5 days starting at day 9 of pregnancy in 100 μl inoculation volume administered by gavage. Mice from the placebo group received 100 μl corn oil only. On day 18 post-mating, pregnant females were separated into individual cages and they gave birth on days 20–22. Subsequently they were allowed to rear their pups for 30 days. Live and stillborn pups were counted, and dead pups were removed. Non-pregnant mice, dams and their offspring were evaluated for clinical signs of disease twice a day from the day of birth to day 30 p.p. Data on the number of dams, non-pregnant mice, and stillborn pups and those dying postnatally were monitored. Non-pregnant mice were euthanzed on day 21 p.i., dams and surviving pups were euthanized on day 30 post-partum. Blood was collected by heart puncture, centrifuged and serum was stored at −20°C. Brain and lung tissue were collected and also stored at −20°C prior to further processing.

### Analysis of biological samples

To quantify the parasite load in brains, DNA extraction was performed according to NucleoSpin® DNA RapidLyse protocol (Macherey-Nagel) employing the standard protocol suitable for animal tissues. The DNA concentrations in all samples were determined using the QuantiFluor dsDNA system (Promega, Madison, WI, USA) according to the manufacturer's instructions and adjusted to 5 ng/μl with sterile DNase-free water. Quantification of parasite loads in the brains of non-pregnant mice and dams was done as described previously (36). Parasite loads in the brain for *N. caninum* were assessed as described previously (25, 28, 37, 38).

### Statistics

Fifty percent inhibitory concentrations (IC_50_s) were calculated after the logit-log transformation of the relative growth (RG; control = 1) according to the formula ln(RG/[1-RG]) = a × ln(drug concentration)+ b and subsequent regression analysis by the corresponding software tool contained in the Excel software package (Microsoft, Seattle, WA, USA). Statistical analysis of the parasite burdens in brains was done using the Kruskal-Wallis test. Comparison of positive and negative animals was performed using the chi-square test. All analyses were performed using the software package R (39).

## Results

### *In vitro* screening of ELQ analogs against transgenic *N. caninum* tachyzoites (Ncβ-Gal) identifies numerous highly active compounds

**Figure 1:**
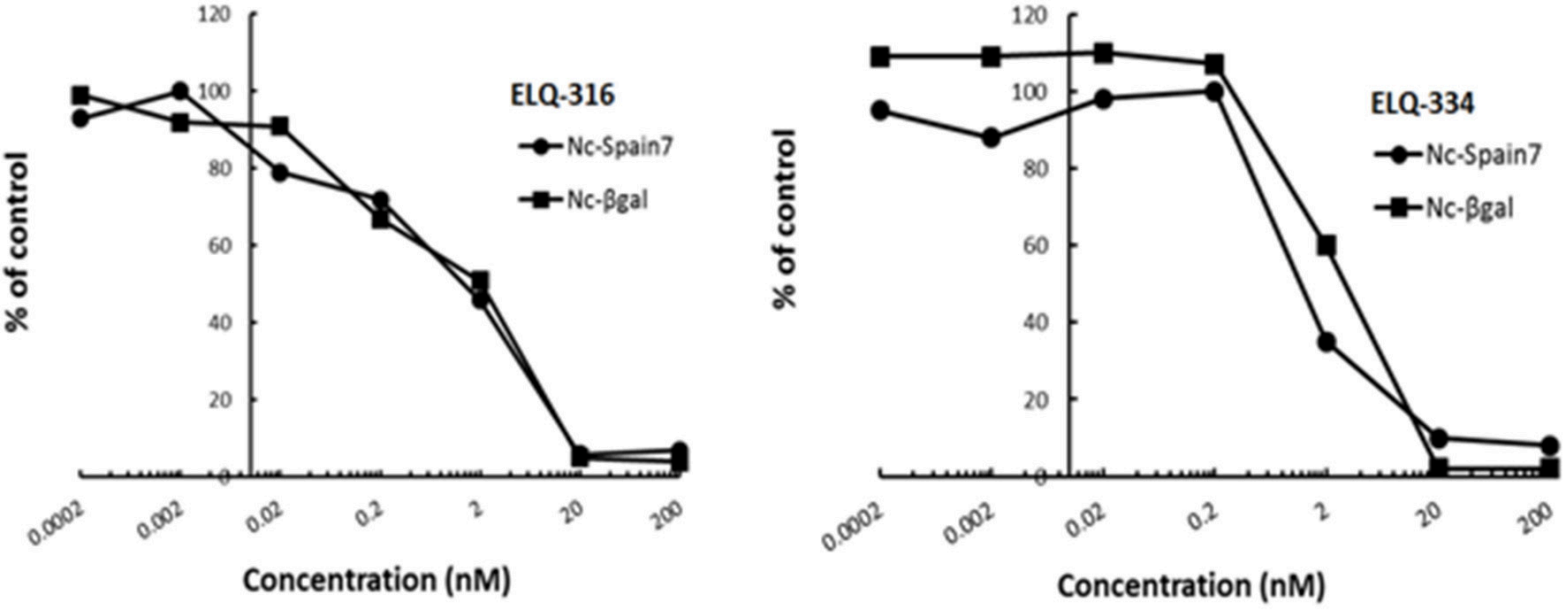
Relative growth curves of Nc-βgal and Nc-Spain7 tachyzoites in response to ELQ-316 and ELQ-334 treatments *in vitro*. Quantification of Nc-β-Gal proliferation was carried out by measuring β-galactosidase activity, the proliferation of Nc-Spain7 was assessed by quantitative real time PCR.

In order to study the effects of the 14 ELQ analogs against *N. caninum* tachyzoite proliferation, HFF were exposed to different concentrations (0–200 nM) of these drugs added concomitantly with infection by Nc-βGal tachyzoites. Since DMSO concentrations above 0.5% induced cytotoxicity in HFF monolayers (data not shown), the maximum ELQ concentration applied to the cultures was 200 nM. The IC_50_ values of the ELQ analogs are shown in Table 1. The lowest IC_50_ was noted for ELQ-136 (0.022 nM), but several other compounds exhibited values below 1 nM, such as endochin, ELQ-121, ELQ-300, ELQ-316, and ELQ-437. IC_50_ values between 1 and 5 nM were noted for ELQ-127, ELQ-271, ELQ-334, ELQ-433, ELQ-434, ELQ-435, and ELQ-436. One compound, ELQ-432, had a higher IC_50_ value (22.5 nM). Since ELQ-316 had been reported earlier to exhibit outstanding efficacy against *T. gondii* tachyzoites and tissue cysts *in vivo* (16), this compound and its prodrug, ELQ-334 (24) were selected for further investigations. The respective inhibition curves for these two compounds are shown in Figure 1.

Since our standardized *in vivo* model for studies on *N. caninum* infection in mice is based on the virulent Nc-Spain 7 isolate (25, 40, 41), the *in vitro* inhibition achieved with ELQ-316 and ELQ-334 was also assessed in HFF infected with Nc-Spain7 tachyzoites, and quantified by real time PCR (Figure 1). Calculation of the IC_50_ values for ELQ-316 in NcSpain7 (IC_50_ = 0.69 nM) closely matched the respective value obtained by using the Ncβ-Gal strain (IC_50_ = 0.66 nM, see Table 1), and similar values for both *Neospora* strains were also obtained for ELQ-334 (Nc-Spain7 IC_50_ = 1.7 nM; Ncβ-Gal IC_50_ = 3.3 nM). Thus, the two *N. caninum* strains exhibit very similar susceptibility toward these two ELQs (Figure 1).

### TEM identified distortion of the mitochondrial matrix, inner membrane and cristae in *N. caninum* tachyzoites as the main ultrastructural alterations induced by ELQ-316 and ELQ-334

**Figure 2:**
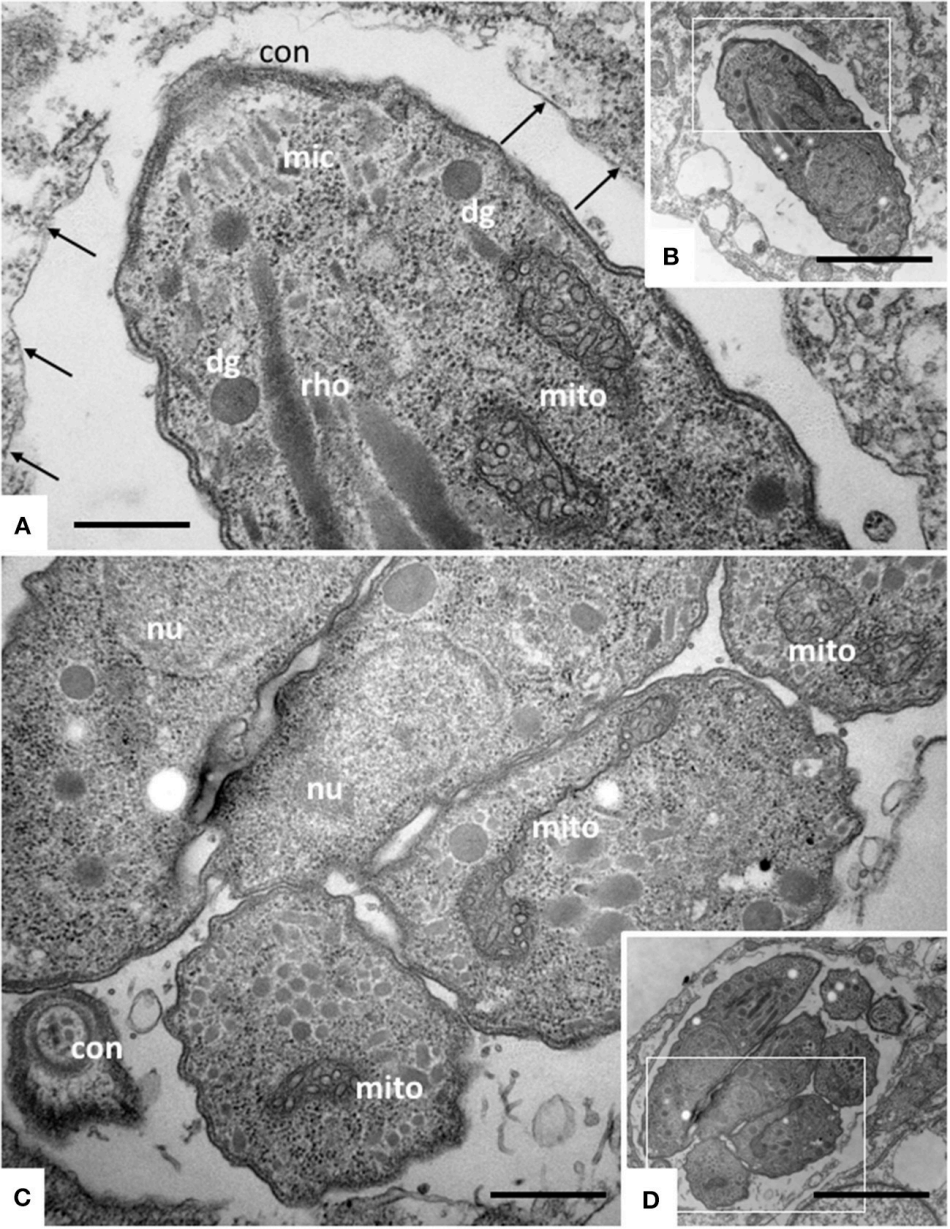
TEM of Nc-Spain 7 tachyzoites cultured in HFF. **(A,B)** Show a single tachyzoites situated within a parasitophorous vacuole, **(C,D)** shows a vacuole containing several parasites undergoing proliferation. The areas framed in **(B,D)** are shown at higher magnification in **(A,C)**, respectively. Note the mitochondrium (mito) with a distinct electron dense matrix and cristae; con, conoid; dg, dense granule; rho, rhoptries; arrows point toward the parasitophorous vacuole membrane. Bar in **(A)** = 0.46 μm; **(B)** = 2.3 μm; **(C)** = 0.54 μm; **(D)** = 2.3 μm.

**Figure 3:**
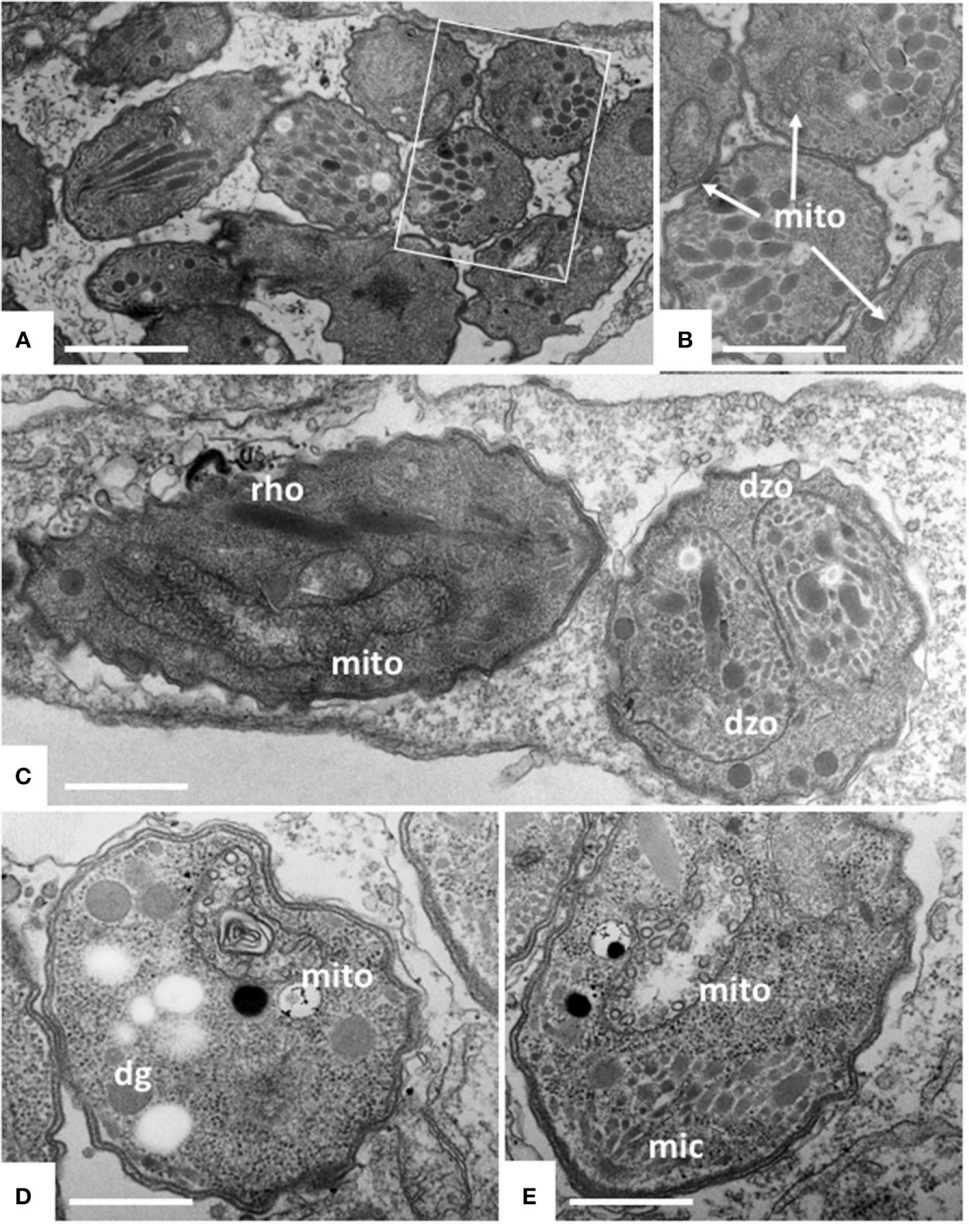
TEM of Nc-Spain7 tachyzoites cultured in HFF and treated with ELQ-334 for 6 h **(A,B)**, 24 h **(C)** and 72 h **(D,E)**. A shows numerous tachyzoites in a parasitophorous vacuole, the framed area is shown at higher magnification in **(B)**. Mitochondria (mito) exhibit slight alterations in some, but not all parasites. Similar situation after 24 h, in some instances parasites forming daughter zoites (dzo) through endodyogeny can be seen. More pronounced alterations in the mitochondrial matrix are seen after 72 h **(D,E)**. Rhoptries (rho), dense granules (dg) and micronemes (mic) do not appear to be affected. Bar in **(A)** = 1.6 μm; **(B)** = 1 μm; **(C)** = 2 μm; **(D,E)** = 0.8 μm.

**Figure 4:**
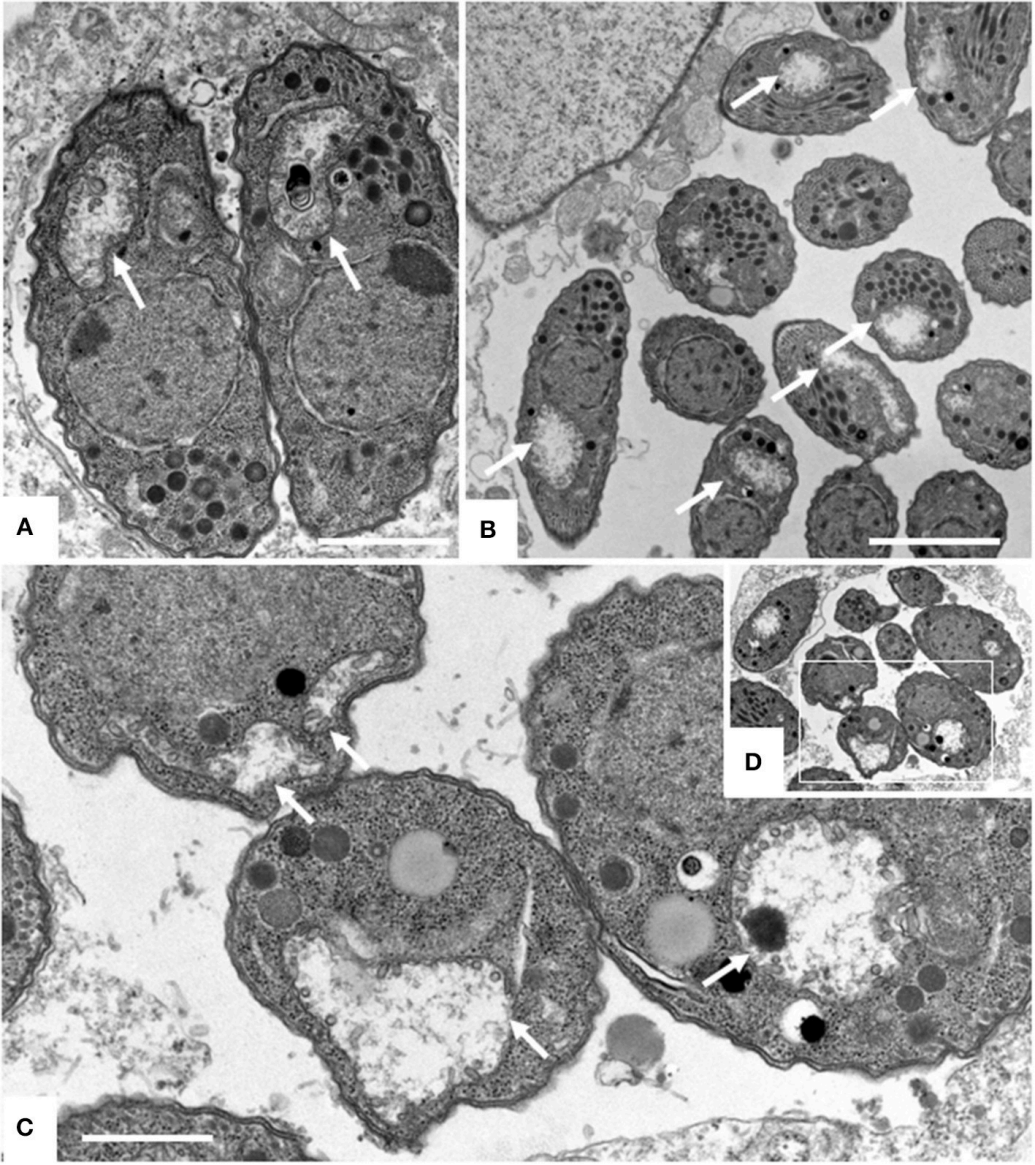
TEM of Nc-Spain7 tachyzoites cultured in HFF and treated with ELQ-316 for 6 h **(A)**, 12 h **(B)** and 48 h **(C,D)**. The framed area in **(D)** is shown at higher magnification in **(C)**. Note that tachyzoites exhibit a dissolved mitochondrial matrix and altered cristae already after 6 h of treatment, and that this aberrant phenotype remains during subsequent timepoints (white arrows). Bars in **(A)** = 1 μm; **(B)** = 1.6 μm; **(C)** = 0.8 μm.

TEM was carried out on *N. caninum* infected HFF exposed to 1 μM ELQ-334 and ELQ-316 at different time-points of treatments ranging from 6 to 72 h. As shown in Figure 2, tachyzoites cultured in the absence of any drugs exhibited the typical features reminiscent for all apicomplexans. Inside their host cell, they were located within a parasitophorous vacuole (PV), surrounded by a parasitophorous vacuole membrane (PVM), and the apical complex containing the conoid, micronemes, rhoptries, and dense granules were clearly visible in longitudinal sections (Figures 2A,B). *N. caninum* tachyzoites contain a single mitochondrion with a tubular structure, of which different parts can be seen in a single section. These mitochondria typically exhibit an electron dense matrix and numerous cristae, clearly discernible by TEM (Figures 2A–D). Upon treatment with 1 μM ELQ-334 (Figure 3) slight alterations in the matrix of the mitochondrion were visible already after 6–24 h (Figures 3A–C). The mitochondrial matrix became less electron dense, primarily in more central areas of the mitochondrion, and alterations in the cristae structure also become evident in some parasites (Figures 3A–C). However, parasites were still proliferating, as evidenced by tachyzoites undergoing endodyogeny (Figure 3C). After 48 h of ELQ-334 treatment (Figures 3D,E), the ultrastructural alterations in the mitochondrial matrix and the cristae became more evident. In some parasites, membrane stacks formed within the interior of the mitochondrion. Similar results were obtained for ELQ-316-treated parasites (Figure 4), although already at 6 h after initiation of treatment, rather massive changes in the mitochondrial matrix could be visualized (Figure 4A), which were also increasingly evident after 24 h (Figure 4B) and 48 h (Figures 4C,D). However, no other structural distortions could be seen in ELQ-334 and ELQ-316 treated tachyzoites. Most notably, the cytoplasmic organization including rhoptries, micronemes and dense granules, remained intact, intracellular parasites appeared to be enclosed in a PV, and other organelles did not seem to be affected to any large extent. Overall, this suggests that ELQs do not induce rapid death of *N. caninum* tachyzoites, but mainly impact on tachyzoite proliferation *in vitro* by primarily acting on the structural integrity of the mitochondrion.

### Long term treatment assays show that ELQ-316 and ELQ-334 do not kill parasites rapidly

HFF infected with Nc-Spain7 tachyzoites, and subsequently underwent continuous treatment with 500 nM ELQ-316 and ELQ-334 for extended periods of time, ranging from 3 to 17 days, after which drug pressure was released and cultures were further maintained without compound. Daily inspection by light microscopy showed that in control cultures devoid of drugs parasites proliferated rapidly, which would lead to host cell lysis and re-infection of neighboring cells latest after 3–4 days post-infection. In contrast, continuous ELQ-treatment for 17 days resulted in strongly impaired proliferation and a delay of host cell lysis, but did not act parasiticidal. Lysis plaques could be observed latest days after removal of the drug (data not shown).

**Figure 5:**
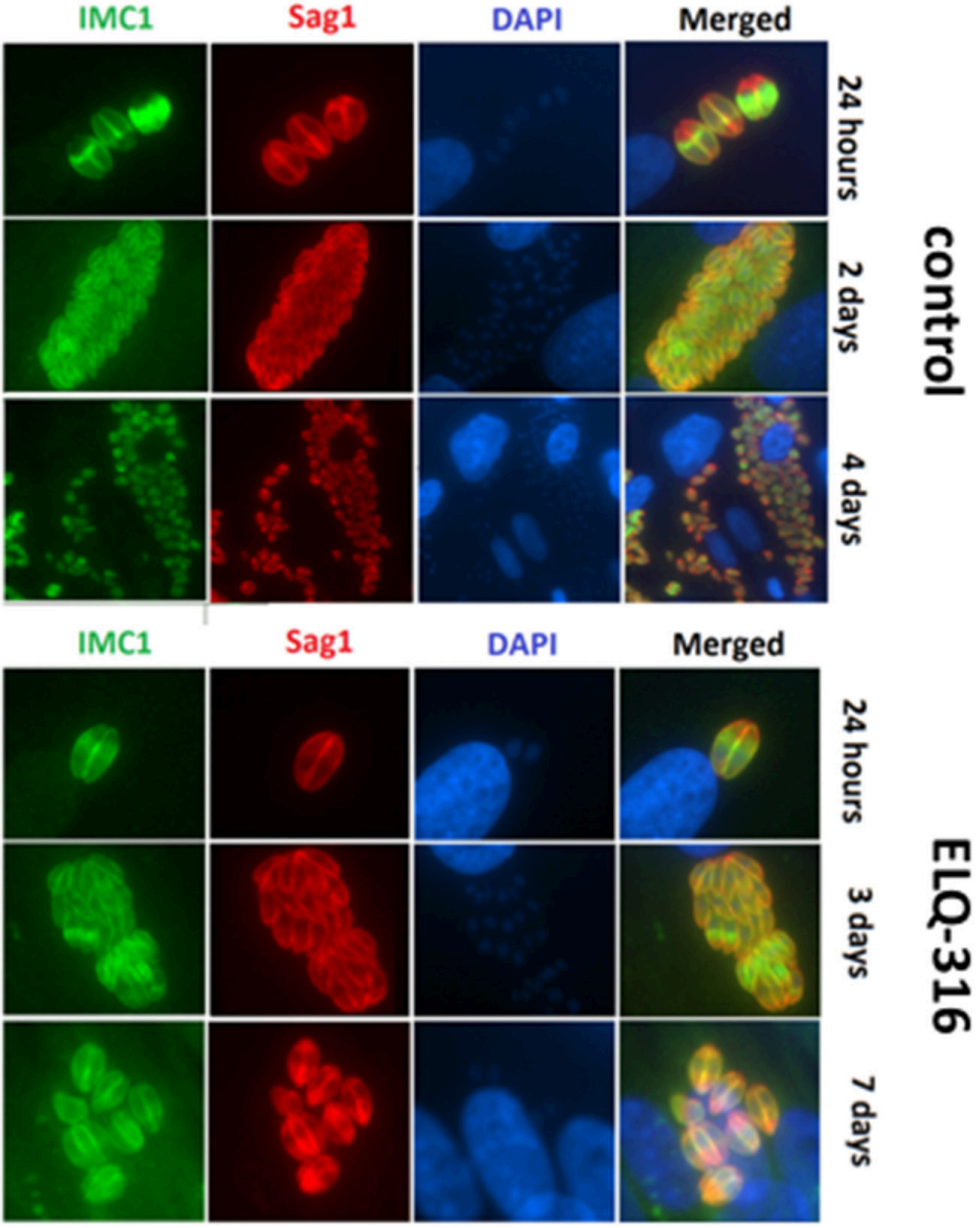
Effects of ELQ-316 against already invaded tachyzoites. Nc-Spain7 tachyzoites were allowed to invade HFF during 3 h, then non-invaded parasites were washed away and cultures were further maintained either in the absence of compounds (control), or 1 μM ELQ-316. Samples were fixed and processed for IF after different timepoints of drug treatment. Specimens were labeled with anti-IMC1 (green) or anti-SAG1 (red). Note that the proliferation inhibitory effects are not very pronounced when treatment is started after host cell invasion.

The inherent capacity of *N. caninum* tachyzoites to undergo proliferation even in the presence of high concentrations of ELQ-316 and ELQ-334 was further assessed by immunofluorescence microscopy employing antibodies directed against NcSAG1 and IMC1. In untreated HFF (Figure 5A) large parasitophorous vacuoles containing numerous tachyzoites were present already after 2 days, closely followed by egress of tachyzoites and subsequent re-infection of neighboring cells. In samples treated with ELQ-316, proliferation of parasites was clearly delayed, and egress was not, or only sporadically, observed (Figure 5B). Similar results were noted for cultures treated with ELQ-334 (data not shown).

Treatment of pregnant mice with ELQ-334 has no adverse effects on pregnancy and confers partial inhibition of vertical transmission of *N. caninum*.

*In vivo* studies in mice were carried out with ELQ-334, which after application in mice is rapidly metabolized to ELQ-316. Due to the increased absorption of ELQ-334 compared to ELQ-316, this leads to approximately six times increased exposure (24).

In a first experiment, we investigated whether ELQ-334 would have an effect on fertility and pregnancy outcome. Results are summarized in Table 2. Daily treatment of pregnant mice (*n* = 6 per group) during days 9–13 of pregnancy with 10 mg/kg/day during 5 days did not affect fertility, nor did this have a negative effect on the number of viable pups. Twenty seven pups were born in the ELQ-334 treatment group and 19 pups in the placebo group that received corn oil only. A single stillbirth occurred in the ELQ-334 treated group. No negative impact on the further development of offspring mice could be detected during 14 days *post-partum*, indicating that this compound was safe for use in pregnant mice.

**Table 2:** Treatment with ELQ-334 does not have significant interference in pregnant BALB/c mice.

**Treatment**	**Fertility**	**Pups per dam**	**Mortality**
Vehicle (corn oil)	4/6	19/4	0/19
ELQ-334	4/6	27/4	1/27

*The number of dams in both experimental groups was equal, and the number of pups in the ELQ-334 treated group was higher than in the vehicle treated group. In the ELQ-334 treated group one pup died in the pre-natal period*.

In the second *in vivo* study, ELQ-334 treatments were applied to mice that were experimentally infected with *N. caninum* tachyzoites on day 7 of pregnancy (results are summarized in Table 3). Thus, mice (*n* = 14 per group) were mated, and during days 9–13 of pregnancy, they were treated with ELQ-334 or corn oil as indicated above. Ten mice gave birth to offspring in the ELQ-334 treated group, 8 in the placebo group, while 4 and 6 mice remained non-pregnant, respectively.

**Table 3:** Effects of ELQ-334 treatments on clinical signs, mortality, fertility, and cerebral Nc-Spain7 infection in non-pregnant mice, dams, and pups.

	**ELQ-334^a^**	**Placebo^a^**
**NON-PREGNANT FEMALES**		
Total no.	4	6
Clinical signs	0/4	0/6
Mortality	0/4	0/6
*N. caninum* PCR positive	3/4	6/6
**DAMS**		
Total no.	10	8
Clinical signs	1/10	0/8
*N. caninum* PCR positive dams	7/10^b^	8/8
**PUPS**		
Litter size	44	34
Neonatal mortality^e^	7/44^c^	15/34
Postnatal mortality^f^	0/37^d^	18/19
*N. caninum* PCR positive pups	19/37^d^	1/1

a * Mice were treated with ELQ-334 in corn oil or with corn oil alone as a placebo control, infected with Nc-Spain7, and sacrified as described. Adults and surviving pups were tested for the presence of N. caninum in their brains quantitative real-time PCR. Pups that had died before the end of the experiment were considered N. caninum positive. Respective numbers of animals in control and ELQ-334-treated groups were compared by chi-square tests*.

b *P > 0.1*.

c *P < 0.025*.

d *P < 0.001*.

e *Proportion of pups born dead or that died within the 2 first days post partum*.

f *Proportion of pups that died during day 3–28 post partum*.

**Figure 6:**
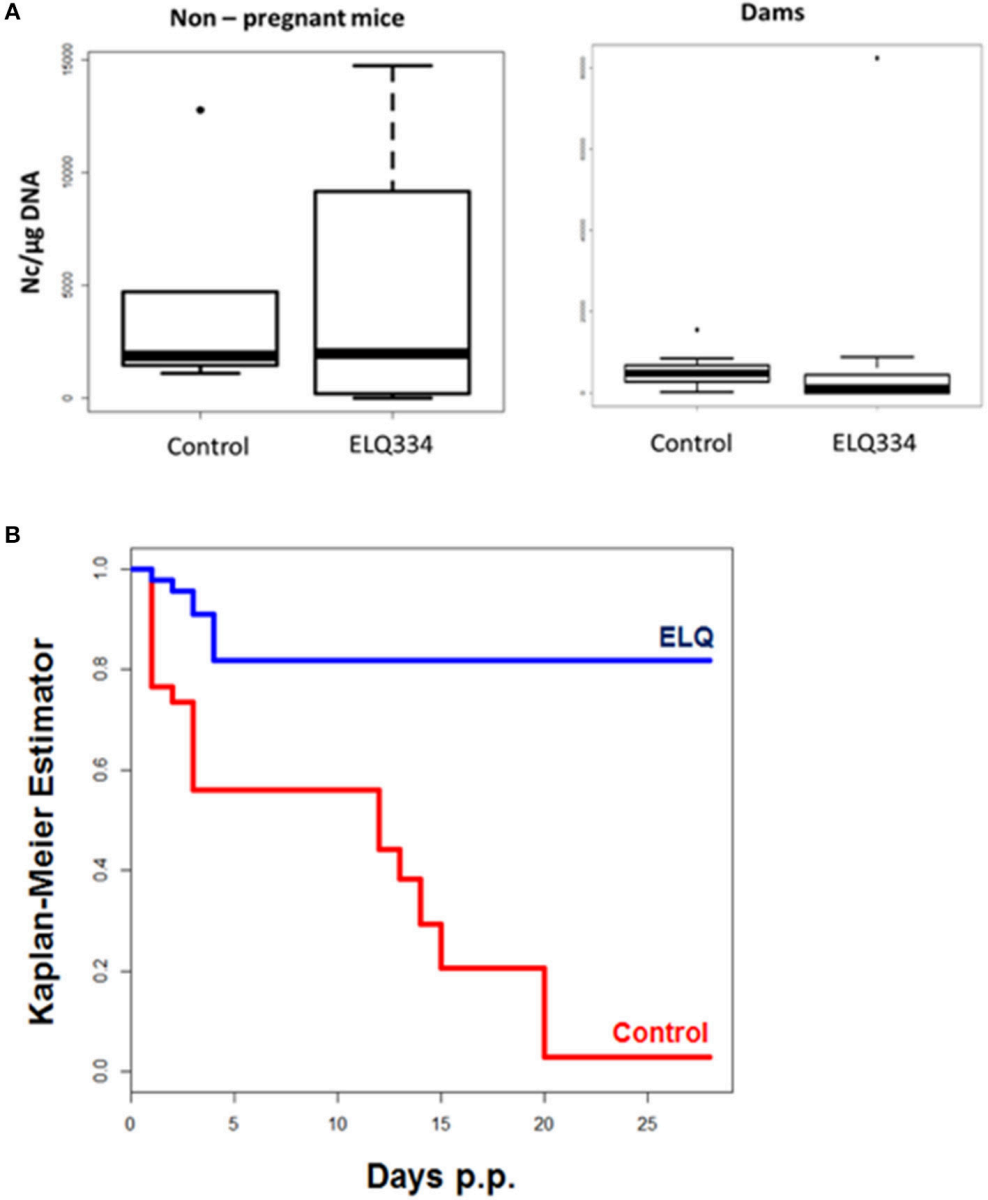
**(A)**. Cerebral parasite load in non-pregnant mice and dams as determined by quantitative real time PCR of brain samples of ELQ-334 treated and placebo-treated control mice. Values are given as number of tachyzoites per μg DNA. There are no significant differences. **(B)**. Kaplan-meier survival curves of pups born from ELQ-334 treated dams and placebo-treated control mice infected with *N. caninum* tachyzoites.

Following infection, none of the adult mice exhibited clinical signs of neosporosis, with the exception of one dam from the ELQ-334 treated group on day 45 p.i. This dam had also a high infection load in the brain. Overall, PCR analysis of brain tissues of dams and non-pregnant mice at the end of the experiment detected parasite DNA in 7 out of 10 dams and in 3 out of 4 non-pregnant mice that were ELQ-334 treated, while all mice in the placebo group were PCR positive no matter whether pregnant or not. Quantification of the cerebral parasite load in the brain samples of the dams and non-pregnant mice in the different treatment groups did not reveal any statistically significant difference between the groups (Figure 6A).

As indicated in Table 3, the 10 dams in the ELQ-334 treatment group gave birth to 44 pups, the 8 dams in the placebo control group gave birth to 34 pups. The pup survival curves are shown in Figure 6B. Seven out of Forty-Four pups (16%) in the ELQ-334 group died within 2 days post-partum (neonatal mortality). However, all 37 remaining pups remained healthy without clinical signs during the 4 weeks follow-up phase. Brain samples of 19 of these 37 pups (51%) tested PCR positive (Table 3). Of the 34 pups that were born the placebo group, neonatal mortality was noted for 15 pups (44%), and postnatal mortality occurred for all but one of the remaining 19 pups (94%). The brain samples of all pups in the placebo group were PCR-positive.

## Discussion

We here report on the *in vitro* efficacy of 14 distinct ELQ analogs against the apicomplexan parasite *N. caninum* in 3-day proliferation inhibition assays, and also show that two of these compounds, ELQ-334 and ELQ-316, induce distinct ultrastructural changes in the parasite mitochondrium. *In vitro* culture of *N. caninum* infected HFF treated with ELQ-334 and ELQ-316 over extended periods of time indicates that these compounds slow down, but do not entirely inhibit tachyzoite proliferation, if treatment is initiated after host cell invasion has occured. They do not kill these parasites after extended continuous treatment at 500 nM over 17 days. Nevertheless, ELQ-334, a prodrug that is metabolized to ELQ-316, exhibits promising safety and efficacy in the pregnant mouse model for *N. caninum* infection by profoundly increasing pup survival and inhibiting vertical transmission by almost 50% at 10 mg/kg.

ELQs have been demonstrated to be efficacious against various protozoan parasites [reviewed in Alday and Doggett (42)]. Like other quinolones, ELQs affect the mitochondrial electron transport chain. The cytochrome *bc*_1_ complex is composed of cytochrome *b*, cytochrome *c*_1_ and the iron-sulfur protein (ISP), which form the central core for electron (and associated proton) transfer. Based on their site of action, inhibitors of the mitochondrial respiratory chain cytochrome *bc*_1_ complex are classified as either Qo or Qi site inhibitors. The Qo site oxidizes ubiquinol and the Qi site reduces ubiquinone. Important currently marketed Qo site inhibitors are atovaquone and buparvaquone. Atovaquone is used as an alternative treatment and prophylactic for toxoplasmosis, and in combination with other drugs for the treatment of malaria and mild to moderate babesiosis (43). Buparvaquone is the only availabe drug for the treatment of *Theileria parva* and *T. annulata* infection in cattle (44). Buparvaquone was shown to exhibit promising *in vitro* activity and *in vivo* efficacy in pregnant mice infected with both, *T. gondii* (45) and *N. caninum* (26). Qi site inhibitors, such as ELQ-316 and other 4(1H)-quinolone-3-diarylethers are not yet in clinical use, but analogs that do not inhibit mamalian cytochrome *bc*_1_ could potentially be useful for treatment in species such as cattle and dogs. Recently ELQ-400 was identified by screening of the MMV (medicines for malaria venture) Pathogen Box as a highly active compound against *N. caninum* tachyzoites *in vitro*, and ELQ-400 also conferred protection against cerebral infection and disease in a neosporosis cerebral infection mouse model (36).

The previously reported IC_50_ values for ELQ-316 and ELQ-271 against *T. gondii* were 0.35 and 5.6 nM, respectively (20). In our assays, the respective values for *N. caninum* Nc-βGal were 0.66 and 2.7 nM. While the initial screening was performed using Nc-βGal, all other studies including mouse experiments were carried out employing a more virulent strain, Nc-Spain7 (40). The IC_50_ values for Nc-Spain7 (0.69 nM for ELQ-316 and 1.72 nM for ELQ-334) and Nc-βGal (0.66 nM for ELQ-316 and 3.33 nM for ELQ-334) are in the same range, which confirms their efficacy and excludes the possibility that these two strains could differ in susceptibility.

Six of the Fourteen ELQs tested in this study, including ELQ-316, exhibited IC_50_s below 1 nM, which represent the lowest values for drug-induced inhibition reported for *N. caninum* so far. The most potent compounds have a fluorine at position 5 on the quinolone core, a structural determinant that is associated with binding at the Qo site (20). This finding is consistent with previous ELQ SAR found in *T. gondii*. Overall, the *in vitro* efficacy against *N. caninum* was similar to *T. gondii* with the exception of ELQ-300, which was 75-fold more potent against *N. caninum* than *T. gondii*. The reason for this difference in activity is not clear as the cytochrome *b* of the two organisms are more than 99% identical. This result raises the possibility that ELQ-300 may have an alternate target in *N. caninum*.

However, it is important to note that a low IC_50_ value observed *in vitro* does not necessarily translate into promising *in vivo* efficacy, since many other aspects of a compound such as absorption, metabolic stability, pharmacokinetic properties, bioavailability, and other parameters play major roles. The most potent compounds identified in these studies have poor oral bioavailability and may be improved by prodrug formulation in a manner similar to ELQ-316 and ELQ-300. Buparvaquone has a reported IC_50_ of 4.9 nM and previous studies have shown greater efficacy in inhibiting vertical transmission in *N. caninum* infected mice than this study of ELQ-334; however, the dose of ELQ-334 was administered at 10 mg/kg in this study compared to the 50 mg/kg dose of buparvaquone (26). Similar to ELQ-334, buparvaquone did not impact significantly on cerebral infection in dams. The bumped kinase inhibitor BKI-1294 with a markedly higher IC_50_ of 40 nM (46) exerted excellent efficacy in *N. caninum* infected mice, massively reduced cerebral infection both in dams and non-pregnant mice, and inhibited vertical transmission to pups by over 90% when given at 50 mg/kg/d for 8 days (25, 46). Artemisone, an artemisinin derivative with enhanced efficacy against malaria displayed an IC_50_ of 20 nM *in vitro*, but was completely ineffective against *N. caninum* infection in the mouse model (26). More recently, novel aza-artemisinins have been synthesized and tested against *N. caninum in vitro*, displaying selective toxicity and IC_50_s of 40 and 110 nM, but they have not yet been assessed *in vivo* yet (47).

As visualized by TEM, ELQ-316 and ELQ-334 had a profound impact on the ultrastructure of the parasite mitochondrion, which already after a short treatment duration (6 h in the case of ELQ-316 and 12–24 h for ELQ-334) lost its characteristic electron dense matrix and cristae, both of which are important for the energy production and synthesis of intermediate metabolites such as pyrimidine. The delayed effect of ELQ-334 could be related to its metabolism to ELQ-316 by cell esterases. There seems to be a marked difference to buparvaquone, where ultrastructural changes upon treatment with 1 μM buparvaquone were visible only after 3 days of treatment (36). Thus, these ELQs exert their activity on the mitochondria at a much earlier time point. As a consequence, proliferation is delayed in the presence of these two drugs, but obviously not entirely inhibited. Clearly, these structural alterations do not automatically imply that all mitochondrial activities are impaired, thus the question how these parasites can overcome this obvious structural defect needs to be addressed in further studies. ELQs block oxidative phosphorylation, thereby causing the relocation of electrons to other biomolecules generating free radicals and reactive oxygen species (ROS) detrimental to the parasite. By the disappearance of structurally intact mitochondria, the parasite could overcome this effect of the drugs, thus energy metabolism is then dependent on glycolysis, and intermediate metabolites need to be scavenged from the host, which in turn would ensure survival, despite impaired proliferation. Similar findings were recently obtained in buparvaquone treated *Besnoitia besnoiti* tachyzoites grown in human fibroblasts, which were found to rapidly adapt to buparvaquone concentrations that were 5,000 times higher than the original IC_50_ (48). TEM also identified a distinct PV and PVM in HFF infected with both, non-treated and drug-treated *N. caninum* tachyzoites, and since the compounds are added concomitantly with the parasites, we conclude that the processes leading to host cell invasion and PV formation were not notably inhibited by these compounds. This is in contrast to ELQ-400, which was identified through *in vitro* screening of the MMV Pathogen Box (30). ELQ-400 had a notably higher IC_50_ (4 nM), but was shown to impact on both, host cell invasion and intracellular proliferation, and the most striking feature that could be observed by TEM was also the dramatic breakdown of the mitochondria (41). In contrast, as suggested by immunofluorescence studies and initiating treatments only after parasites have invaded their host cells, ELQ-316 and ELQ-334 act mainly on host cell invasion rather than intracellular proliferation. ELQ-400 has not yet been evaluated in the pregnant neosporosis mouse model, but was shown to negatively impact on cereberal infection in non-pregnant Balb/c mice, being more efficient than ELQ-334 in preventing cerebral infection (30). The higher efficacy of ELQ-400 could be explained by a recent study suggesting that ELQ-400 targets both Q_o_ and Q_i_ sites on cytochrome *b* (19) or may be related to pharmacokinetics.

The result for ELQ-334 in the pregnant mouse model are promising. Under the conditions used, the compound was safe and did not interfere in pregnancy outcome, and there are no issues that would indicate toxicity. ELQ-334 treatment in *N. caninum* infected mice reduced loss of offspring by partially inhibiting neonatal mortality and completely preventing postnatal mortality. In addition, vertical transmission was prevented in approximately 50% of the surviving pups. A compound that blocks vertical transmission completely has not been identified to date, but other drugs with similar or even better efficacy haven been reported, such as toltrazuril, BKIs and buparvaquone (25, 41, 46, 49, 50). In this respect, ELQ-334 could be potentially combined with another quinolone such as the Q_o_ site inhibitors atovaquone (51) or buparvaquone, to achieve dual site inhibition and probably increased efficacy, or with another compound of different mode of action. Proof of concept for such a combination therapy was provided in immunodeficient mice experimentally infected with *B. microti*, where radical cure was achieved using a combination of ELQ-334 and atovaquone at a dosage of 5 mg/kg, with no recrudescence of babesiosis during a time span of 122 after discontinuation of therapy (24). Future investigations should focus on such combination approaches.

## Author contributions

AH, JD, and MR conceived the study and participated in its design. AH coordinated the biological assays part of the study. NA, VB, JM, PW, AA-M, and MR performed cell culture, *in vitro* assays and inhibition studies. NA and MR performed real time PCR. NA, AA-M, PW participated in the *vivo* studies in mice. SP, AN, MR, and JD designed, synthesized and provided ELQ compounds. NA and AH did the interpretation of results, and NA, AH, and JD wrote the manuscript.

### Conflict of interest statement

The authors declare that the research was conducted in the absence of any commercial or financial relationships that could be construed as a potential conflict of interest.
